# Liver-Directed Therapies Can Be More Than “Local Tumor Control”

**DOI:** 10.1007/s00270-026-04514-3

**Published:** 2026-06-29

**Authors:** Federico Collettini, Geert Maleux

**Affiliations:** 1 Department of Radiology, Charité University Medicine Berlin, Charitéplatz 1, 10117 Berlin, Germany; 2 Berlin Institute of Health (BIH), Anna-Louisa-Karsch-Straße 2, 10178 Berlin, Germany; 3 Department of Radiology, University Hospitals KU Leuven, Herestraat 49, 3000 Louvain, Belgium

The CHOPIN trial, recently reported by van den Hoek and colleagues in The Lancet Oncology, is an investigator-initiated, single-center, open-label, randomized phase 2 study testing whether percutaneous hepatic perfusion (PHP) with melphalan gains incremental benefit when combined with dual immune checkpoint inhibition (ipilimumab + nivolumab) in patients with previously untreated, unresectable liver-only or liver-dominant metastatic uveal melanoma [1]. CHOPIN reports a large, statistically significant improvement in progression-free survival with combination therapy (adjusted HR 0.34; 1-year PFS 54.7% vs 15.8%) and a marked increase in objective response rate (76.3% vs 39.5%). Overall survival (OS) also favored the combination arm (adjusted HR 0.39), although the study was not primarily powered for OS. The trade-off is substantial: grade ≥ 3 treatment-related adverse events (AE) occurred in 82% of patients in the combination group vs 41% with PHP alone, with both increased perfusion-related toxicity and immune-related AEs, including one treatment-related death attributed to immunotherapy-associated “triple M” syndrome, comprising myasthenia gravis, myositis, and myocarditis [1]. More specifically, grade 3 or higher perfusion-related adverse events were observed more frequently with the combination regimen than with PHP alone (71% vs 32%). The biological explanation for this increase remains unclear. The authors suggest, however, that the addition of ipilimumab and nivolumab may have intensified PHP-associated toxicity by enhancing immune activation. In particular, immune checkpoint blockade may have augmented systemic inflammatory responses, promoting cytokine release and macrophage activation, and thereby contributing to greater hematological toxicity and hemophagocytic manifestations [1]. Accordingly, the excess severe perfusion-related toxicity may reflect a biologic interaction between the physiological stress induced by PHP and immune checkpoint inhibitor-driven immune upregulation, rather than merely an additive increase in typical immune-related adverse events.

Although any interpretation of CHOPIN must be tempered by the intrinsic limitations of a single-center, open-label, randomized phase 2 study with a comparatively modest sample size, constraints that precluded a robust assessment of potential abscopal effects in extrahepatic disease and rendered the observed overall survival signal suggestive rather than definitive, for the interventional radiology community the trial nonetheless stands as a pivotal proof-of-concept that liver-directed therapy can be more than “local tumor control”: it can be positioned as a systemic treatment partner designed to reshape the hepatic tumor microenvironment. Beyond uveal melanoma, CHOPIN strengthens a broader immune-oncologic thesis: liver metastases are disproportionately influential, both as a life-limiting “organ failure” driver and as an immunologic niche that can attenuate checkpoint inhibitor efficacy [2, 3]. Clinical-translational evidence shows that patients with liver metastases may have poorer responses and shorter PFS to anti–PD-1 therapy in melanoma and NSCLC and exhibit reduced CD8+ T-cell density at tumor margins; importantly, reduced CD8 infiltration was also observed in distant extrahepatic metastases, suggesting systemic immune consequences rather than a purely local phenomenon. Mechanistic work in mouse models and patient cohorts has provided a plausible explanation: liver metastases can recruit immunosuppressive macrophages that promote antigen-specific T-cell apoptosis in the liver, effectively “siphoning” tumor-reactive T cells and reducing systemic antitumor immunity; liver-directed radiotherapy in these models reshaped the hepatic immune microenvironment and restored immunotherapy efficacy [4]. These concepts generalize to immunotherapy-resistant disease contexts. In microsatellite-stable metastatic colorectal cancer, a secondary analysis of the randomized Canadian Cancer Trials Group CO.26 trial found that patients without liver metastases had improved PFS and higher disease control with Durvalumab+Tremelimumab, whereas liver metastases were associated with worse outcomes, supporting the need to stratify future immunotherapy trials by liver metastasis status [5].

CHOPIN provides unusually persuasive randomized evidence that a liver-directed interventional oncology platform can materially improve disease control when paired with dual checkpoint inhibition in a disease historically resistant to immunotherapy. The study can be read as a clinical experiment aligned with the “liver metastasis as an immunologic gatekeeper” hypothesis: high-dose regional chemotherapy induces tumor cell death and antigen release in the liver while dual checkpoint blockade reduces inhibitory signaling, potentially increasing the probability that systemic antitumor T-cell responses are generated or sustained. CHOPIN itself frames this as a mechanistic rationale, and it plans translational analyses of blood and tissue to clarify immune interactions. However, CHOPIN simultaneously cautions that liver-directed therapy plus ICI can produce additive or supra-additive toxicity, and it did not establish a robust abscopal effect in extrahepatic disease (due largely to small numbers). Therefore, for multi-cancer extrapolation, the most defensible inference is not “PHP + ICI controls systemic metastases,” but rather: optimizing liver disease control may be a prerequisite for maximizing the value of systemic immunotherapy in liver-dominant metastatic states, a premise already supported by cross-tumor immunotherapy outcome data.

In conclusion, the CHOPIN encourages us to view our locoregional therapies not simply as local weapons, but as dynamic and potentially transformative components of a more ambitious oncologic vision. Beyond local tumor control, our locoregional therapies may reshape the biological and clinical trajectory of the illness, opening the door to broader therapeutic effects and new strategic opportunities within multidisciplinary cancer care. Along these lines, it is important to recognize that the diversity and heterogeneity of locoregional therapies in interventional oncology represent both a major opportunity and a considerable challenge. The wide spectrum of available treatment platforms, including chemoembolization, radioembolization, hepatic arterial infusion, and thermal as well as non-thermal ablative techniques, may exert distinct and treatment-specific effects on the tumor microenvironment and systemic antitumor immunity. These modalities are therefore likely to be associated with unique immunological signatures, shaped by differences in their mechanisms of tumor cell death, vascular effects, inflammatory responses, antigen release, and cytokine induction. Further research is therefore needed to more precisely characterize these immunomodulatory profiles and to determine how they may be optimally exploited within rational combination strategies.
